# Direct-current potential in assessment of puerperas severity

**DOI:** 10.1186/2197-425X-3-S1-A344

**Published:** 2015-10-01

**Authors:** I Zabolotskikh, T Musaeva, E Denisova

**Affiliations:** Kuban Medical University, Krasnodar, Russian Federation

## Introduction

At the present time, the clinicians utilize many score systems for assess the patient's state severity. The sensitivity and specificity of all the systems are vary depending on the score systems and the different groups of patients. Registration of the direct-current potential (DCP) in a forehead-palm lead allow the clinicians to identify three different functional state of the human body.

## Objectives

To estimate the prognostic significance of the APACHE II, APACHE III, SAPS II, SAPS 3 and SOFA scoring systems in puerperas with different DC potential levels.

## Methods

Retrospective study 367 puerperas with severe sepsis was performed. The median age was 26.0 (22,0-34,0) years.

All the patients were assessed using the APACHE II, APACHE III and SAPS II, SAPS 3 and SOFA scoring systems. Score systems were compared using a Hosmer-Lemeshow test and AUROC (Area Under Receiver Operator Curve). Three different functional states: compensated, subcompensated and decompensated were specified depending on the DCP level. Then, calculation of severity using the score systems were modified depending on the functional state. The score systems were compared using the Hosmer-Lemeshow test and AUROC (Area Under Receiver Operator Curve) again.

## Results

The area under the ROC curve was 0.79 for APACHE II, 0.85 for APACHE III, 0.81 for SAPS II, 0.83 for SAPS 3 and 0.84 for SOFA. The Hosmer-Lemeshow goodness-of-fit test H statistic revealed poor performance for APACHE II and SAPS II systems. The sensitivity and specificity of APACHE II, APACHE III, SAPS II, SAPS 3 and SOFA scoring systems increases, when the functional state of the patient was taken into consideration. The area under the ROC curve was 0.83 for APACHE II, 0.89 for APACHE III, 0.84 for SAPS II, 0.88 for SAPS 3 and 0.91 for SOFA. But the Hosmer-Lemeshow goodness-of-fit test H statistic also revealed poor performance for APACHE II scoring system.

## Conclusions

The functional state of the body can be determined by the DCP level. The functional state of the patient significantly influences the outcome. The functional state of the patient may be used for improvement of mortality prognosis.
